# Protective Effects of the Ketogenic Diet on Cognitive Impairment Induced by Status Epilepticus in Rats: Modulation of Neuroinflammation Through the NF‐κB Signaling Pathway

**DOI:** 10.1002/pdi3.70013

**Published:** 2025-06-23

**Authors:** Wandi Wang, Lingman Wang, Chunxue Jiang, Shengxuan Zhang, Chen Tan, Liqiong Peng, Ran Ding, Bing Tian, Xiaojie Song, Li Jiang

**Affiliations:** ^1^ Department of Neurology National Clinical Research Center for Child Health and Disorders Ministry of Education Key Laboratory of Child Development and Disorders Chongqing Key Laboratory of Child Neurodevelopment and Cognitive Disorders Children's Hospital of Chongqing Medical University Chongqing China

**Keywords:** epilepsy, ketogenic diet, neuroinflammation, nuclear factor‐kappa B (NF‐κB) pathway, status epilepticus

## Abstract

Epilepsy is a chronic neurological disorder characterized by abnormal synchronized neuronal discharges, leading to cognitive dysfunction. The ketogenic diet (KD) has shown promise as an effective treatment for drug‐resistant epilepsy (DRE), reducing seizures and improving cognitive and behavioral outcomes in patients. However, the precise neuroprotective mechanisms are not fully understood. This study aimed to investigate the effects of KD on cognitive impairment and hippocampal neurocircuit damage in rats with status epilepticus (SE), with a focus on a nuclear factor‐kappa B (NF‐κB) signaling. SE was induced using pilocarpine, and rats were assigned to KD and control groups. After 7 and 20 days of KD treatment, cognitive function was assessed using the elevated plus‐maze, Morris water maze, novel object recognition, and Y‐maze tests. Hippocampal tissue was analyzed for structural damage of neurocircuit. NF‐κB pathway activation was evaluated by western blot and immunofluorescence. Results indicated that KD significantly improved cognitive performance and reduced hippocampal damage. Additionally, KD inhibited NF‐κB pathway activation, evidenced by decreased levels of NF‐κB, p‐IκB, and proinflammatory cytokines. These findings suggest that KD may alleviate cognitive deficits and hippocampal damage by modulating the NF‐κB signaling, providing insights into its neuroprotective mechanisms and potential as an alternative treatment for epilepsy.

## Graphical abstract

Ketogenic diet has therapeutic effects on neurocircuitry damage and cognitive impairment due to status epilepticus in rats by modulating the NF‐κB signaling pathway.

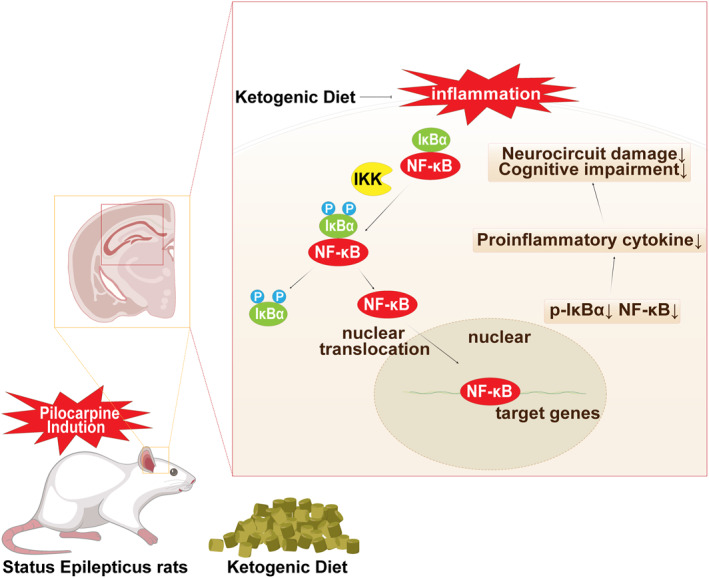

## 1 Introduction

Epilepsy is a neurological disorder marked by recurrent seizures, which result from hypersynchronous excitatory electrical discharges in a group of neurons within the brain. As one of the most prevalent chronic neurological diseases, it has a global incidence ranging from 6.38‰ to 7.60‰, affecting over 70 million individuals worldwide, 30% of whom require treatment with two or more antiepileptic drugs concurrently [1]. When seizures are not adequately controlled, the condition is classified as drug‐resistant epilepsy (DRE) [2]. Frequent seizures not only increase the risk of sudden death in epileptic patients [3] but also cause significant impairments in various cognitive functions, including memory, language, executive function, attention, and information processing speed [4]. These impairments profoundly impact both the daily life and work of affected individuals. With the advancement of the biopsychosocial medical model, many researchers have emphasized that the management of epilepsy should extend beyond seizure control to also address comorbidities such as cognitive impairment [5].

The classical ketogenic diet (KD), originally formulated in the 1920s as a metabolic therapy, is characterized by a high‐fat (approximately 90% of total calories) low‐carbohydrate composition with balanced protein and essential micronutrients. This nutritionally complete formulation maintains physiological homeostasis without compromising somatic development while demonstrating therapeutic efficacy across diverse pathological conditions, including neurological disorders (e.g., epilepsy and neurodegenerative diseases) and oncological applications [6]. KD has been extensively utilized and proven effective in treating DRE in children since the 1920s, demonstrating superior efficacy compared to current antiepileptic drugs. This suggests that KD may act through a general protective mechanism for the brain [7, 8]. Recent studies have further substantiated the neuroprotective effects of KD, particularly in cases of brain injury and neurodegenerative diseases [9]. Additionally, accumulating evidence indicates that KD positively influences cognitive function in various conditions. It has been shown to ameliorate cognitive dysfunction in patients with Alzheimer's disease (AD), Parkinson's disease, and autism [10, 11], while also enhancing vocabulary comprehension, information processing speed, alertness, and attention in epilepsy patients [12]. Furthermore, KD has been reported to improve sleep quality and alleviate mental health issues such as depression, anxiety, and fatigue [13]. However, the precise mechanisms underlying KD's neuroprotective and anticonvulsant effects remain insufficiently understood. Some studies suggest that the beneficial effects of KD may be mediated through the reduction of oxidative stress and inflammation [7, 14, 15].

Neuroinflammatory responses have been linked to the progression of AD in experimental models. The anti‐inflammatory effects of KD are thought to involve the inhibition of the nuclear factor‐kappa B (NF‐κB) signaling pathway, a key regulator of inflammatory gene expression. By attenuating NF‐κB activation, KD may reduce the production of proinflammatory cytokines and chemokines [16]. Studies have demonstrated that KD decreases NF‐κB's nuclear translocation and DNA‐binding activity, leading to the suppression of inflammatory gene expression. This inhibition of NF‐κB signaling results in decreased production of molecules, such as interleukin‐1 beta (IL‐1β), tumor necrosis factor‐alpha (TNF‐α), interleukin‐6 (IL‐6), and other cytokines and chemokines, involved in inflammation. However, further research is required to fully elucidate the specific mechanisms through which KD modulates NF‐κB and its role in the diet's anti‐inflammatory effects [17]. Despite the growing interest, few studies have focused on whether KD can improve cognitive function and the underlying mechanisms. Therefore, our study aims to investigate whether KD ameliorates neurocircuit damage and cognitive function in a lithium chloride‐pilocarpine rat model of SE and whether it exerts anti‐inflammatory effects by decreasing the activity of the NF‐κB signaling pathway.

## 2 Materials and Methods

### 2.1 Experimental Animals

All animal procedures were conducted in strict accordance with the National Research Council's Guidelines for the Care and Use of Laboratory Animals and the ARRIVE Guidelines, which are designed to ensure transparency, reproducibility, and integrity in the reporting of our research. The protocols were rigorously reviewed and approved by the Ethics Committee, Children's Hospital of Chongqing Medical University, under protocol CHCMU‐IACUC20240628009, thereby complying with both international and national regulations regarding animal experimentation.

Male juvenile Sprague–Dawley (SD) rats (postnatal day 21, P21) were supplied by the Experimental Animal Center of Chongqing Medical University. The animals were housed under controlled environmental conditions with a 12 h light/dark cycle, temperature maintained at 22–24°C, and relative humidity of 50%–60%. Certified experimenters with animal research qualifications identified rat genders through external genital morphology. The study strictly adhered to animal welfare guidelines to minimize suffering and optimize the use of experimental animals.

### 2.2 Establishment of the SE Model

In this experiment, a pilocarpine‐induced SE rat model was employed. The experimental procedures were conducted as follows. Male SD rats, aged 21 days postnatally, were randomly selected for the study. A total of 150 P21 male SD rats were initially enrolled, with 70 randomly assigned to the normal control group (Group N) and 80 allocated to the SE modeling group (SE group). The SE group received an intraperitoneal injection of lithium chloride (127 mg/kg, Sigma, Saint Louis, Missouri, US), followed by pilocarpine (50 mg/kg, Sigma, Saint Louis, Missouri, US) 18–20 h later. Seizure activity was evaluated using Racine's grading scale. Rats that did not exhibit convulsive seizures or failed to reach Racine grade 4 within 30 min were administered additional pilocarpine (10 mg/kg). Animals not attaining Racine stage 4 seizures after three supplemental pilocarpine doses (10 mg/kg) were excluded from further analysis (exclusion rate: 12.5%, 10/80 rats). To minimize pilocarpine‐induced side effects, atropine sulfate (1 mg/kg, Kingyork Group, Tianjin, China) was administered intraperitoneally 15 min after seizure onset, followed by chloral hydrate (300 mg/kg) 30 min later to terminate the convulsions. In the normal control group (Group N), physiological saline of equivalent volume was substituted for pilocarpine, whereas lithium chloride and atropine were administered at identical dosages and schedules as those applied to the SE group. This protocol ensured single‐variable control to eliminate potential confounding effects of lithium chloride and atropine on experimental rats. Among the 70 SE rats meeting inclusion criteria, 8 required supplemental pilocarpine administration. During subsequent standard housing, 7 SE rats died (4 from the supplemental pilocarpine subgroup and 3 from the nonsupplemented subgroup), resulting in 63 surviving rats (4 with supplemental pilocarpine and 59 without) for downstream experimental analyses. The epileptic rats were closely monitored and provided with supportive care, including the maintenance of body temperature, clearance of respiratory secretions, and oral gavage with sugar and saline until self‐feeding resumed.

### 2.3 Experimental Grouping and Dietary Intervention

**FIGURE 1:**
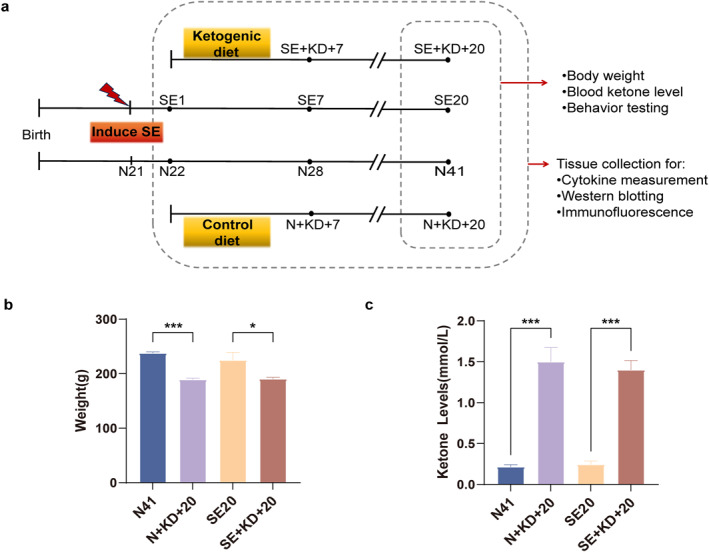
Effects of KD on body weight and blood ketone levels in different groups of rats. (a) Experimental procedure flowchart: On the first day after SE, rats in the SE and N groups were fed either a KD or a normal diet for 7 or 20 days. Body weight and blood ketone levels were measured in the dietary intervention and control groups after 20 days of dietary intervention, followed by behavioral testing. Tissue specimens were collected at designated time points across all experimental groups. (b) Effect of KD intervention on body weight in rats (*n* = 7). (c) Effect of KD intervention on blood ketone levels in rats (*n* = 7). Values are expressed as the mean ± SEM. *: *p* < 0.05 and ***: *p* < 0.001. KD, ketogenic diet.

Male p21 immature SD rats were randomly assigned to four groups: normal control (N), control + ketogenic diet (N + KD), status epilepsy (SE), and SE + ketogenic diet (SE + KD). Rats that were injected with lithium chloride‐pilocarpine to successfully induced SE were randomly assigned to the SE and SE + KD groups. Rats that were not injected with lithium chloride‐pilocarpine and did not exhibit seizures were randomly assigned to the N and N + KD groups. Subsequently, the N and SE groups were maintained on a standard diet, whereas the SE + KD and N + KD groups were fed a KD containing approximately 10% protein, 89.9% fat, and 0.1% carbohydrates (D10070801, ReadyDietech, Shenzhen, Guangdong, China). Food was provided ad libitum during the dietary intervention, and identical housing conditions were maintained across all groups. The KD was replaced daily and at 7 and 20 days of intervention. The experimental groups and procedures are depicted in Figure 1a. Following the 20‐day intervention period, body weights of all experimental groups were systematically recorded and blood ketone levels were quantified through tail vein sampling to validate the metabolic efficacy of the ketogenic intervention.

### 2.4 Behavioral Testing

#### 2.4.1 Elevated Plus Maze (EPM) Test

A typical behavioral test for assessing anxiety‐like behavior in rats, the EPM test is based on the rats' innate fear of open elevated spaces [18]. Each arm of the apparatus measures 50 × 10 cm, with two open arms and two enclosed arms. The open arms do not have side walls, whereas the enclosed arms are encircled by opaque walls that are 40 cm high. A center platform, 10 × 10 cm, connects the arms, creating a plus‐shaped structure, that is, 50 cm above the ground. Rats spent 5 min exploring freely after being positioned one at a time on the center platform, facing one of the enclosed arms. The duration in open arms, open arm access frequency, and total arm transition events were systematically recorded. An arm entry was operationally defined as complete displacement of all four paws into the designated arm. Behavioral data acquisition utilized the ANY‐maze video tracking system (Stoelting Co., Wood Dale, Illinois, USA). Open arm dwell time percentage was derived from the equation: (open arm duration/total experimental duration) × 100%. Correspondingly, open arm access proportion was determined via (open arm entries/total arm entries) × 100%.

#### 2.4.2 Morris Water Maze (MWM) Test

The MWM serves as a standardized paradigm for evaluating spatial cognition in rodent models, requiring subjects to orient using environmental cues to identify a submerged platform [19]. At 20 days post‐SE induction, eight randomly assigned rats per cohort were subjected to MWM testing for the cognitive assessment. Initial habituation involved 60 s water exposure in a circular arena (150 cm diameter and 80 cm height; 14 cm platform) divided into cardinal quadrants (N, E, S, and W). Spatial acquisition trials commenced on day 2 with five consecutive training days (four daily trials). Starting positions alternated daily between N‐E‐S‐W and W‐S‐E‐N sequences, with platform stabilization in the S quadrant. Unsuccessful navigation attempts (≥ 60 s) resulted in guided platform placement with 15 s retention. Postacquisition, platform removal facilitated spatial probe testing through two 60 s N quadrant releases. Behavioral parameters were quantified as follows: (1) mean S quadrant occupancy duration and (2) platform zone crossings. The ANY‐Maze tracking system (Stoelting Co., Wood Dale, Illinois, USA) captured trajectory patterns, specifically measuring preplatform target quadrant dwell time and platform crossing frequency.

#### 2.4.3 Novel Object Recognition (NOR) Test

The NOR test is a widely used behavioral assay for evaluating hippocampus‐dependent recognition memory. This test is based on the principle that animals spend more time exploring a novel object than a familiar one [20]. The test is conducted in an open empty box (80 × 80 × 80 cm^3^), containing two objects of similar height and volume, but distinct in shape and appearance. During the familiarization phase, mice were exposed to two identical objects, placed equidistantly in opposite corners, for 10 min. The box was then cleaned with 75% ethanol. Twenty‐four hours later, the animal was returned to the box for the second phase of the test. In the second phase, one of the familiar objects was replaced with a novel object of comparable mass and the animal was allowed to explore freely for 5 min. NOR data were analyzed using the ANY‐maze video tracking system (Stoelting Co., Wood Dale, Illinois, USA). The discrimination ratio (DR) was calculated using the formula: ([time spent exploring the novel object]/[time spent exploring the novel object + time spent exploring the familiar object]) × 100%.

#### 2.4.4 Y‐Maze Spontaneous Alternation Test

Spontaneous alternation in the Y‐maze, a widely used test for assessing spatial working memory, is based on the innate exploratory behavior of rats [21]. The test is conducted in a Y‐shaped maze with three arms of equal dimensions, constructed from opaque plastic. The arms, each measuring 35 × 5 × 15 cm^3^, were arranged at 120° angles to one another and labeled A, B, and C. Rats were introduced at the end of arm B and allowed to explore freely all three arms for 8 min. The alternation rate was determined by recording the number of arm entries and triads. Total arm entries were recorded manually by a treatment‐blinded observer or automatically using the ANY‐maze system (Stoelting Co., Wood Dale, Illinois, USA). A sequential entry into three distinct arms was defined as an alternation. An entry was recorded when all four limbs of the rats were fully within an arm. The alternation rate (entry into a different arm from the previous two) was calculated using the formula: (alternations/[total arm entries − 2]) × 100%.

#### 2.4.5 Cytokine Measurement

Commercial enzyme‐linked immunosorbent assay (ELISA) kits (JiangLai Biology, Shanghai, China) were used to measure proinflammatory cytokines (IL‐6 [JL20896], IL‐1β [JL20884], and TNF‐α [JL13202]) in the hippocampal supernatants of rat brain homogenates. Hippocampal tissue was homogenized in phosphate buffered saline (PBS) and centrifuged at 12,000 × g for 10 min at 4°C to collect the supernatant. Samples were introduced into capture antibody‐precoated ELISA microplates and sustained under refrigerated conditions (4°C) for 16 h immunoligand binding. Following three cycles of stringent washing with PBS‐T buffer, horseradish peroxidase (HRP)—conjugated secondary antibodies were administered and maintained at ambient thermal conditions (25 ± 1°C) for 60 min epitope recognition. Chromogenic reaction was triggered by 3,3′,5,5′‐tetramethylbenzidine (TMB) substrate infusion, with signal amplification terminated via acidic termination reagent. Optical density measurements at 450 nm wavelength were executed using a RT‐6100 microplate reader (Rayto, Shenzhen, Guangdong, China). Cytokine concentrations were calculated from standard curves.

#### 2.4.6 Immunofluorescent Staining

The expression of NeuN, NF200, MBP, and NF‐κB in hippocampal neurons was quantitatively analyzed. After the rats were anesthetized with isoflurane inhalation, they were perfused sequentially with 0.9% saline (50–100 mL) followed by 4% paraformaldehyde (50 mL). After dissection, the brain was fixed in 4% paraformaldehyde for 24 h, followed by gradient dehydration in sucrose solution (30%, then 15%). The brain was sectioned into 30‐μm thick coronal frozen sections using a cryostat microtome. Following blocking, the sections were incubated overnight at 4°C with mouse anti‐NeuN antibody (1:100, MAB377; Sigma‐Aldrich, St. Louis, Missouri, USA), rabbit anti‐NF200 antibody (1:200, Zenbio, Chengdu, Sichuan, China), mouse anti‐MBP antibody (1:1000, SMI99; BioLegend, San Diego, California, USA), and mouse anti‐NF‐κB p65 antibody (1:200, Zenbio, Chengdu, Sichuan, China). After incubation, DAPI (1:2000, Leigen, Beijing, China) and appropriate secondary antibodies were applied for secondary staining in the darkroom. Images were captured using a confocal microscope (Nikon A1R, Tokyo, Japan) at 20× magnification, with the same exposure time for all sections.

#### 2.4.7 Western Blotting

Hippocampal specimens were microdissected under cryogenic conditions and immediately cryopreserved in liquid nitrogen. Total protein lysates were prepared from hippocampal tissue using the BB‐3101 extraction system (Bestbio, Shanghai, China), with subsequent protein normalization conducted via the Pierce BCA Assay Kit (UB276924; Thermo Fisher Scientific, Waltham, Massachusetts, USA). Equivalent protein aliquots (30 μg/lane) were resolved through sodium dodecyl sulfate–polyacrylamide gel electrophoresis (SDS‐PAGE) and electrophoretically transferred to polyvinylidene difluoride (PVDF) membranes using semidry methodology. Postblocking with 5% nonfat milk/tris‐buffered saline with tween 20 (TBST) for 60 min, membranes were subjected to antigen‐antibody interaction with specified primary immunoreagents under controlled refrigeration (4°C) for 16 h immunoblotting: mouse anti‐NF‐κB p65 antibody (1:2000, Zenbio, Chengdu, Sichuan, China), rabbit anti‐IκB antibody (1:2000, Zenbio, Chengdu, Sichuan, China), rabbit anti‐p‐IκB antibody (1:2000, Zenbio, Chengdu, Sichuan, China), rabbit anti‐MBP antibody (1:8000; GeneTex, Irvine, California, USA), mouse anti‐GAD antibody (1:1000, Zenbio, Chengdu, Sichuan, China), and mouse anti‐GAPDH (1:50000, Proteintech, Wuhan, Hubei, China). Following TBST washing, the membrane was incubated with horseradish peroxidase (HRP)‐conjugated secondary antibodies (1:5000, ZEN BIO, Chengdu, Sichuan, China). Signal detection was performed using enhanced chemiluminescence (MA0186, Meilunbi, Dalian, Liaoning, China) and visualized using a Bio‐Rad Imaging System (Bio‐Rad Laboratories, Hercules, California, USA). Protein expression levels were normalized to glyceraldehyde 3‐phosphate dehydrogenase (GAPDH) and analyzed using the Image Lab 6.0 software (Bio‐Rad Laboratories, Hercules, California, USA).

#### 2.4.8 Statistical Analysis

Quantitative data were computationally processed through GraphPad Prism 9.0 and presented as the mean ± standard error of the mean (SEM). One‐way analysis of variance (ANOVA) was performed, followed by Tukey–Kramer post hoc tests to determine statistical differences between groups. Statistical significance was set at *p* < 0.05, with * indicating *p* < 0.05, ** indicating *p* < 0.01, and *** indicating *p* < 0.001.

## 3 Results

### 3.1 Effects of 20‐day KD on Body Weight and Blood Ketone Levels in Rats

After 20 days of KD intervention, rats in each group were weighed and found to have significantly lower body weights in the N + KD + 20 and SE + KD + 20 groups than in the KD nonintervention groups (N41 and SE20 groups; *F* (3, 24) = 10.19, *p* < 0.05, and Figure 1b). Measurement of ketone body levels in the tail vein blood of rats from the SE20 group revealed no significant differences in blood ketone levels between the N41 group which did not undergo dietary intervention (*p* > 0.05). However, after 20 days of dietary intervention, ketone body levels in KD‐fed rats (SE + KD + 20 and N + KD + 20 groups) were significantly higher than those in the control groups (N41 and SE20) (*F* [3, 24] = 43.09, *p* < 0.001, and Figure 1c), confirming ketosis.

### 3.2 KD Ameliorates Cognitive Deficits in the SE Rat

**FIGURE 2:**
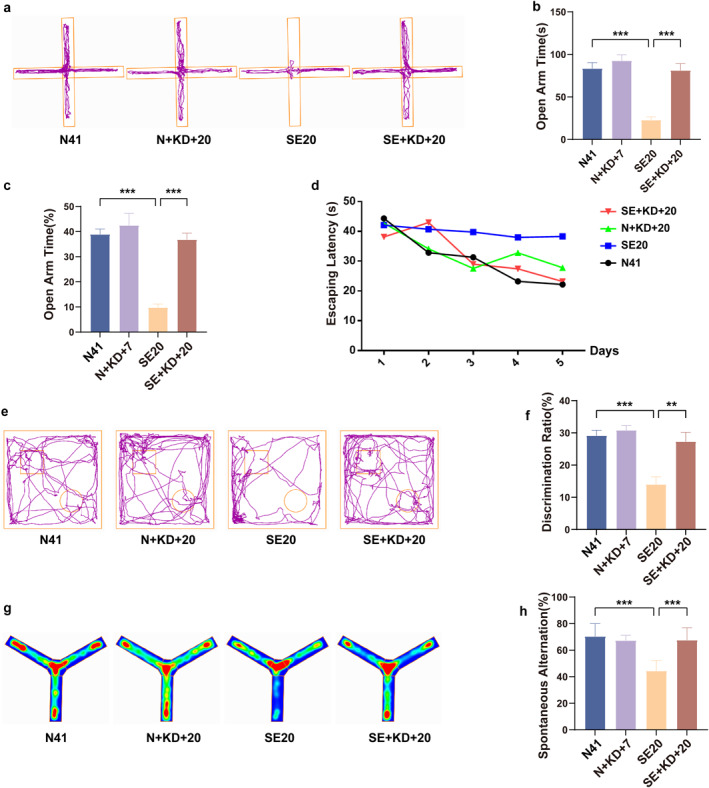
Behavioral testing. (a) Representative activity trajectories of the elevated plus maze experiments (*n* = 8). (b) Time spent in open arms. (c) Percentage of time spent in open arms during the EPM experiments. (d) Escape latency in the Morris water maze test (*n* = 7). (e) Representative activity trajectories of the novel object recognition experiments (*n* = 8). (f) Discrimination ratio for rats in the novel object recognition experiments. (g) Representative heat maps of the Y‐maze experiments (*n* = 8). (h) Spontaneous alternation in Y‐maze experiments. Values are expressed as the mean ± SEM. **: *p* < 0.01 and ***: *p* < 0.001.

We utilized the EPM, MWM, NOR, and Y‐maze tests to investigate the effects of the KD on cognitive and behavioral deficits induced by SE in rats (Figure 2). In the EPM test, the time spent in the open arms (*F* [3, 28] = 22.91, *p* < 0.001, and Figure 2a,b) and the proportion of time spent in the open arms (*F* [3, 28] = 25.01, *p* < 0.001, and Figure 2c) were significantly reduced in the SE20 group compared to the N41 group. In contrast, the SE + KD + 20 group showed a significant increase in both measures compared to the SE20 group. In the MWM test, no significant differences in the average escape latency were observed among the groups during the acquisition trials from Day 1 to Day 5 (*p* > 0.05 and Figure 2d). In the spatial probe test, the SE group displayed significantly fewer platform crossings and spent less time in the target quadrant compared to the control group (*p* < 0.01, Table 1), whereas no significant differences were found between the SE + KD + 20 and SE groups. In the NOR test, the discrimination ratio in the SE20 group was significantly reduced compared to the N41 group, whereas the SE + KD + 20 group demonstrated a significant increase in the discrimination ratio after 20 days of KD treatment compared to the SE20 group (*F* [3, 28] = 12.23, *p* < 0.01, and Figure 2e,f). In the Y‐maze test, the spontaneous alternation rate in the SE20 group was significantly lower than that in the N41 group, whereas the SE + KD + 20 group exhibited a significant increase in spontaneous alternation after 20 days of KD treatment compared to the SE20 group (*F* [3, 28] = 17.90, *p* < 0.001, and Figure 2g,h). Across all four experiments, there were no statistically significant differences between the N + KD + 20 and N41 groups (*p* > 0.05 and Table 1).

**TABLE 1:** Number of platform crossings and time spent in the target quadrant for every group (mean ± SEM).

Group	*n*	Number of platform crossings (*n*)	Time in the target quadrant (s)
N41	7	2.200 ± 0.547	18.55 ± 3.618
N + KD + 20	7	2.180 ± 0.807	18.95 ± 3.12
SE20	7	1.000 ± 0.578**	14.48 ± 3.136**
SE + KD + 20	7	1.920 ± 0.736	19.28 ± 4.513
		*p* = 0.001	*p* = 0.002

^∗∗^ *p* < 0.01 compared with the control group.

### 3.3 KD Reduces Neuronal Loss in the SE Rat

**FIGURE 3:**
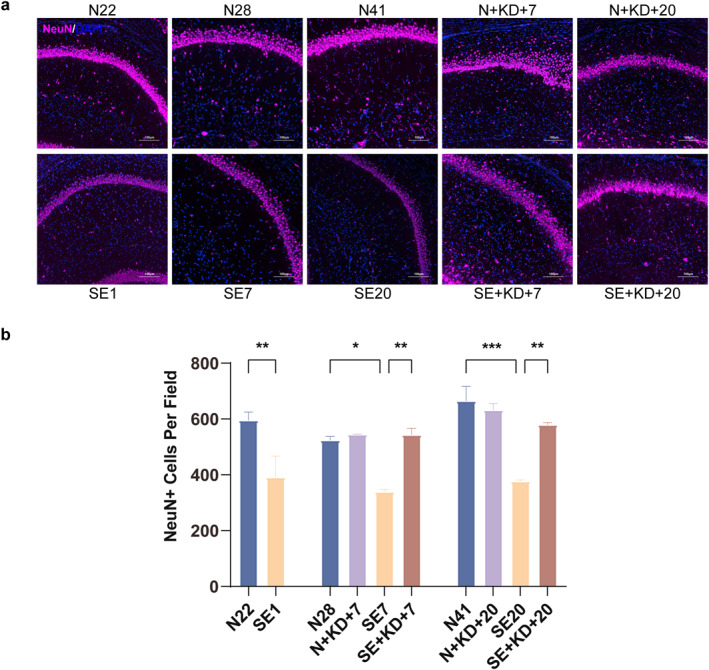
Images showing immunofluorescence staining for NeuN in the hippocampus. (a) Typical images showing immunofluorescence staining of NeuN in the hippocampal CA1 region (NeuN: purple, DAPI: blue). Scale bar: 100 μm. (b) Number of NeuN‐positive neurons in the DG region of the hippocampus. Values are expressed as the mean ± SEM (*n* = 3). *: *p* < 0.05, **: *p* < 0.01, and ***: *p* < 0.001.

We investigated changes in neuronal counts in the hippocampal CA1 region following SE induction and KD treatment using immunofluorescence. Compared to the N22, N28, and N41 group, the SE1, SE7, and SE20 group exhibited a significant reduction in the number of NeuN‐positive cells. In contrast, the SE + KD group showed a significant increase in NeuN‐positive cell counts after 7 or 20 days of KD treatment compared to the SE group (*F* [9, 20] = 11.44, *p* < 0.01, and Figure 3a,b). No significant difference in NeuN‐positive cell counts was observed between the N + KD group and the N group.

### 3.4 KD Alleviates Myelin Damage in the SE Rat

**FIGURE 4:**
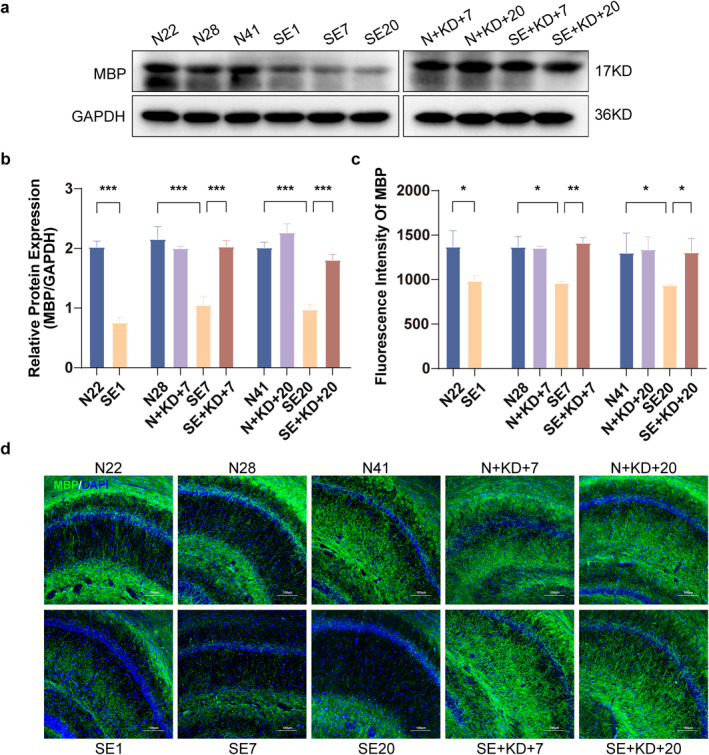
Western blot results and immunofluorescence staining showing MBP levels in the hippocampus at different time points after SE. (a) Representative western blot images of MBP (*n* = 6). (b) Quantitative analysis of MBP levels. (c) Quantitative analysis of MBP expression in the hippocampal CA1 region (*n* = 3). (d) Typical images showing immunofluorescence staining of MBP in the hippocampal CA1 region (MBP: green, DAPI: blue). Scale bar: 100 μm. Values are expressed as the mean ± SEM. *: *p* < 0.05, **: *p* < 0.01, and ***: *p* < 0.001.

The expression levels of myelin basic protein (MBP) in the CA1 region of rat hippocampus were analyzed using immunofluorescence staining and western blotting. Western blotting results showed that MBP expression was significantly decreased in the SE1, SE7, and SE20 group compared to the N22, N28, and N41 group, indicating myelin degradation or damage. Furthermore, MBP expression in the SE + KD group was significantly elevated after 7 or 20 days of KD treatment compared to the SE group (*F* [9, 50] = 21.80, *p* < 0.001, and Figure 4a,b). Immunofluorescence analysis revealed that MBP fluorescence intensity was significantly reduced in SE1, SE7, and SE20 of the SE group compared to that of N22, N28, and N41 of the control group. In contrast, the SE + KD group exhibited a significant increase in MBP fluorescence intensity after 7 or 20 days of KD treatment, approaching the levels observed in the N group (*F* [9, 20] = 7.269, *p* < 0.05, and Figure 4c,d). No significant differences in MBP expression were found between the N + KD group and the N group, excluding the possibility that the KD alone affects MBP expression.

### 3.5 KD Mitigates Axonal Injury in the SE Rat

**FIGURE 5:**
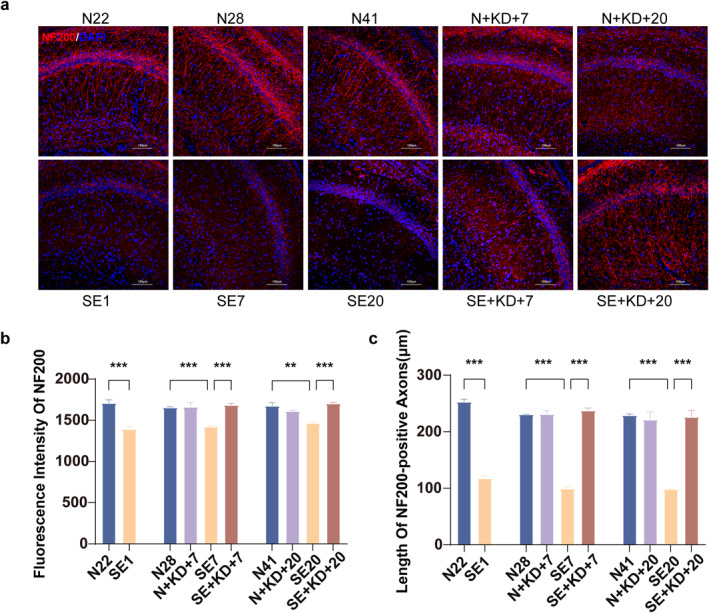
Images showing immunofluorescence staining for NF200 in the hippocampus. (a) Typical images showing immunofluorescence staining of NF200 in the hippocampal CA1 region (NF200: red, DAPI: blue). Scale bar: 100 μm. (b) Quantitative analysis of NF200 expression in the hippocampal CA1 region. (c) Length of NF200‐positive axons. Values are expressed as the mean ± SEM (*n* = 3). **: *p* < 0.01 and ***: *p* < 0.001.

Neurofilament 200 (NF200) was labeled by immunofluorescence staining to assess axonal growth, repair, and damage in the hippocampal CA1 region (Figure 5a). The results showed that NF200 fluorescence intensity and NF200‐positive axonal length were significantly reduced in SE1, SE7, and SE20 of the SE group compared to N22, N28, and N41 in the N group. In contrast, the SE + KD group exhibited significantly increased NF200 fluorescence intensity (*F* [9, 20] = 16.49, *p* < 0.001, Figure 5b) and NF200‐positive axonal length (*F* [9, 20] = 243.5, *p* < 0.001, Figure 5c) after 7 or 20 days of KD treatment, approaching the levels observed in the control group. No significant differences were found between the N + KD group and the N group.

### 3.6 KD Decreases Proinflammatory Cytokine Levels in the SE Rat

**FIGURE 6:**
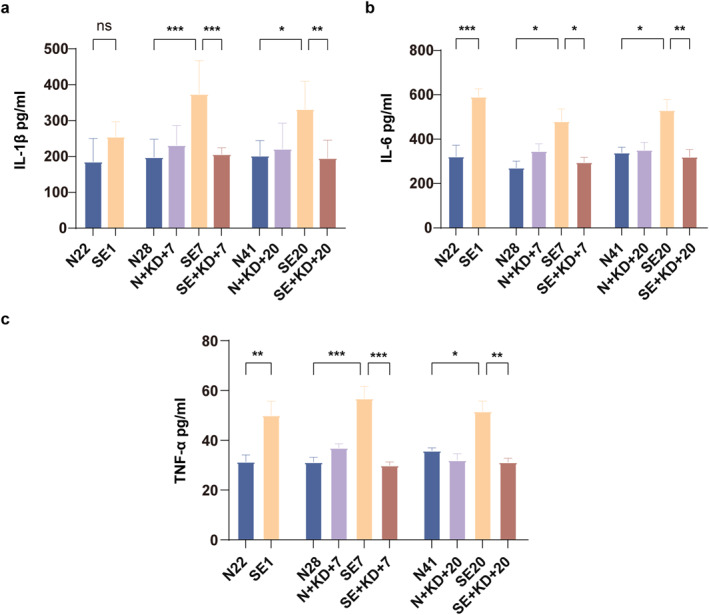
ELISA results showing proinflammatory levels in the hippocampus after SE. (a–c) Levels of IL‐1β, IL‐6, and TNF‐α in the hippocampus were measured by enzyme‐linked immunosorbent assay (*n* = 6). Values are expressed as the mean ± SEM. *: *p* < 0.05, **: *p* < 0.01, and ***: *p* < 0.001.

We measured the levels of IL‐1β, IL‐6, and TNF‐α in the hippocampal region using ELISA. The results showed that compared to N22, N28, and N41 in the control group, the levels of IL‐6 and TNF‐α were significantly increased in SE1, SE7, and SE20 of the SE group, and compared to N28 and N41 in the control group, the levels of IL‐1β was significantly increased in SE7 and SE20 in the SE group. After 7 or 20 days of KD treatment, the levels of IL‐1β (*F* [9, 50] = 6.767, *p* < 0.01, and Figure 6a), IL‐6 (*F* [9, 50] = 8.173, *p* < 0.05, and Figure 6b), and TNF‐α (*F* [9, 50] = 10.35, *p* < 0.01, and Figure 6c) were significantly reduced in the SE + KD group compared to the SE group. The cytokine levels did not differ significantly between the N + KD and N groups (*p* > 0.05).

### 3.7 KD Inhibits NF‐κB Signaling Pathway Activation Following SE

**FIGURE 7:**
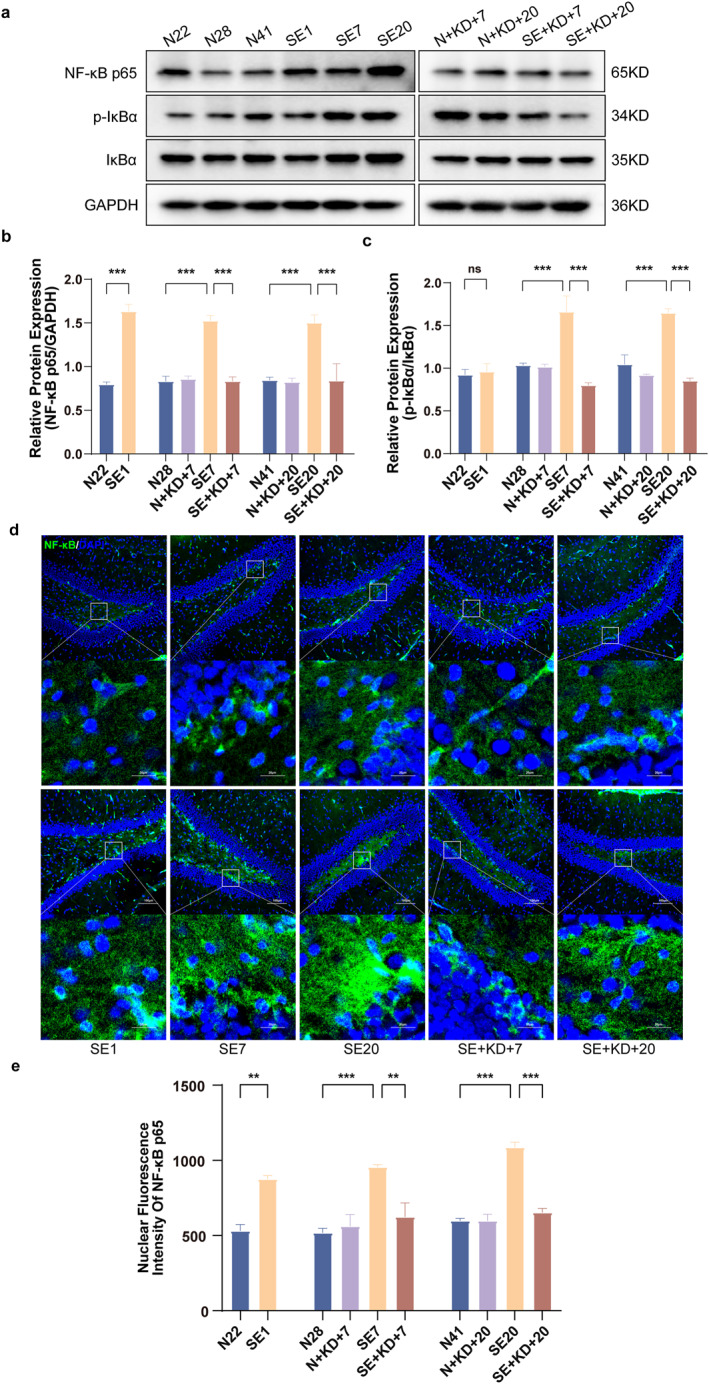
Western blot results showing NF‐κB p65, p‐IκBα, and IκBα levels in the hippocampus after SE, along with immunofluorescence quantification of NF‐κB p65 nuclear translocation in the hippocampal DG region. (a) Representative western blot images (*n* = 6). (b, c) Quantitative analysis of NF‐κB p65 and p‐IκBα/IκBα levels. (d) Representative images showing immunofluorescence staining of NF‐κB p65 in the hippocampal DG region (*n* = 3) (NF‐κB p65: green, DAPI: blue). Scale bar: 100 and 20 μm. (e) Quantitative analysis of nuclear NF‐κB p65 expression in the hippocampal DG region. Values are expressed as the mean ± SEM. **: *p* < 0.01 and ***: *p* < 0.001.

Western blot analysis was performed to assess the expression of NF‐κB p65, p‐IκB, and IκB in the hippocampus (Figure 7a–c). The results showed that compared to N22, N28, and N41 of the control group, the expression of NF‐κB p65 was elevated in SE1, SE7, and SE20 of the SE group and the expression of phosphorylated inhibitor of kappa B/inhibitor of kappa B (p‐IκB/IκB) was increased inSE7 and SE20 of the SE group. Compared to the SE group, the expression of NF‐κB p65 (*F* [9, 50] = 17.71, *p* < 0.001, and Figure 7b) and p‐IκB/IκB (*F* [9, 50] = 14.34, *p* < 0.001, and Figure 7c) was significantly reduced in the SE + KD group after 7 or 20 days of KD treatment. No significant difference was observed between the N + KD and N groups. The expression of NF‐κB p65 in the nucleus of the hippocampal dentate gyrus (DG) region was detected using immunofluorescence (Figure 7d,e). The results showed that compared to N22, N28, and N41 of the control group, the expression of NF‐κB p65 in the nucleus of the hippocampal DG region was elevated in SE1, SE7, and SE20 of the SE groups. Compared to the SE group, the expression of NF‐κB p65 in the nucleus was significantly reduced in the SE + KD group after 7 or 20 days of KD treatment (*F* [9, 20] = 17.80, *p* < 0.01, Figure 7e). No significant statistical difference was observed between the N + KD group and the N group.

## 4 Discussion

Despite the approval of more than 15 new antiseizure medications (ASMs) over the past three decades, approximately 30%–40% of individuals with epilepsy are diagnosed with DRE [22]. For these individuals, the KD may represent a promising therapeutic option for achieving seizure freedom or reducing seizure frequency. In the absence of glucose, the KD stimulates the synthesis of ketone bodies, providing an alternative energy source for the brain. Previous studies have indicated that dietary therapy promotes cognitive development in children with DRE [23]. Although limited published evidence is available, the potential benefits of ketone bodies and associated nutrients in mitigating neurodegenerative diseases and cognitive decline have gained increasing recognition [24].

Increasing evidence suggests that the loss of learning and memory functions in epilepsy patients is linked to various pathological mechanisms, including reduced neurogenesis, aberrant migration of newly formed neurons, abnormal axonal and synaptic reorganization, and persistent inflammation, which can affect both gray and white matter [25, 26]. These pathological changes contribute to hippocampal neuronal loss, axonal sprouting, and synaptic plasticity impairments, thereby disrupting information processing and transmission, ultimately leading to cognitive dysfunction [27]. The hippocampus is fundamentally involved in core cognitive processes, with well‐established roles in learning and memory formation, and contributes to higher‐order functions including decision‐making. In our study, we observed a significant reduction in the number of neurons in the hippocampal CA1 region, shortened hippocampal axons, and a marked decrease in the level of MBP, a key component of the myelin sheath, following the induction of SE. Additionally, behavioral testing revealed impaired learning and memory abilities in these rats 20 days after SE. However, after 7 or 20 days of KD treatment, both the pathological changes and cognitive dysfunction were notably alleviated.

When and how did these pathological changes occur? It is well‐established that the pathological progression of epileptogenesis can be divided into three phases. The initial phase (acute phase) is characterized by the excitotoxic effects of glutamate, leading to rapid neuronal death. This is followed by the second phase (latent phase), during which apoptosis, inflammatory processes, and neuroprotective responses are activated. In the third phase (chronic phase), the brain undergoes long‐term reorganization including synaptic plasticity, axonal sprouting, aberrant neural circuitry, and epileptogenesis [28]. These pathological changes occurring during epileptogenesis may be associated with alterations in brain plasticity and could contribute to cognitive impairments. An increasing body of evidence indicates that neuroinflammation plays a pivotal role in the entire process of brain cell injury, epileptogenesis, and associated neurological complications [29]. Furthermore, KD has been shown to modulate inflammatory pathways by upregulating anti‐inflammatory proteins, such as nucleotide‐binding leucine‐rich repeat‐containing protein 3 (NLRP3) and C‐terminal binding proteins (CtBPs), thereby potentially reducing both systemic‐ and neuroinflammation [30, 31, 32]. As our previous study has shown, classically activated microglia (M1) and alternatively activated microglia (M2) underwent variations throughout the stages of epileptogenesis and the M1‐associated cytokine IL‐1b significantly increased during the acute stage after SE and was maintained at a high level for nearly 3 weeks, finally dropping to control levels by the 28th day [33]. In order to clarify the mechanism by which KD improves hippocampal damage induced by seizures, we further examined the inflammatory cytokines and NF‐κB signaling pathway.

NF‐κB is a crucial nuclear transcription factor present in nearly all cells, responding to various stimuli such as stress, cytokines, free radicals, and bacterial or viral antigens. It is a key regulator of neuroinflammation and immune responses in the central nervous system, particularly during M1 polarization of activated microglia, where it promotes the release of inflammatory cytokines. Normally, NF‐κB remains inactive in the cytoplasm through binding to its inhibitor, I‐κB. Upon I‐κB degradation or phosphorylation, NF‐κB is released, translocates to the nucleus, and initiates the transcription of downstream inflammatory factors, thereby modulating the inflammatory response. The NF‐κB/Rel family includes five members: p65 (RelA/NF‐κB3), p50 (NF‐κB1), p52 (NF‐κB2), Rel (cRel), and RelB, with p65 being the most closely associated with microglial activation [34]. Activation of NF‐κB leads to the transcription of proinflammatory cytokines such as TNF‐α, IL‐6, and IL‐1β [35]. Numerous studies have demonstrated the critical role of NF‐κB signaling in cognitive impairment associated with diseases such as multiple sclerosis, attention deficit disorder, Parkinson's disease, and diabetes‐related cognitive dysfunction. For instance, Wang et al. showed that regulatory T cells mitigate myelin loss and improve cognitive function by suppressing microglial pyroptosis through the TLR4/MyD88/NF‐κB pathway in a demyelination model [36]. In our study, the expression of NF‐κB‐related proinflammatory cytokines (TNF‐α, IL‐6, and IL‐1β) increased during the acute phase of SE and persisted into the chronic phase, alongside NF‐κB pathway activation. However, after 7 or 20 days of KD intervention, the levels of these proinflammatory cytokines returned to baseline and NF‐κB activation was suppressed. These findings suggest that KD alleviates cognitive dysfunction following SE by inhibiting the NF‐κB pathway.

Neuroinflammation and immune activation are central to the pathogenesis of epileptogenesis and its associated cognitive dysfunction. Increasing evidence suggests that the KD may play a modulating role in these processes. A critical aspect of neuroinflammation is the activation of microglia, which has been widely studied in the context of neurodegenerative diseases. Recent studies have shown that ketone bodies, particularly β‐Hydroxybutyrate (BHB), can influence the phenotype of microglia, promoting a shift from the proinflammatory M1 phenotype to the anti‐inflammatory M2 phenotype. This transition is thought to mitigate neuroinflammation and provide neuroprotective effects [37]. In addition to modulating microglial activation, the KD has been shown to suppress the production of proinflammatory cytokines and chemokines, which are key mediators of neuroinflammation and neuronal injury. By dampening these inflammatory signals, the KD may contribute to the resolution of chronic inflammation and its neurotoxic consequences [31]. Moreover, the KD influences astrocyte function, another pivotal component of neuroinflammation. Recent evidence suggests that ketone bodies modulate astrocyte activation and polarization, potentially enhancing their neuroprotective roles while limiting their proinflammatory contributions. This regulation further supports the resolution of neuroinflammation [38, 39]. Beyond its direct effects on neuroimmune cells, the KD also exhibits antioxidant properties, which help neutralize reactive oxygen species (ROS) and reduce oxidative stress. By mitigating oxidative damage to cells and tissues, the KD may protect against inflammation‐driven neuronal injury [40]. Additionally, emerging evidence suggests that the KD can alter the gut microbiota, which plays a crucial role in regulating immune function. By promoting the growth of beneficial bacteria and suppressing pathogenic microorganisms, the KD may exert indirect immunomodulatory effects through the gut–brain axis, further influencing neuroinflammatory pathways [41]. In summary, the immunomodulatory effects of the KD are multifaceted, involving complex interactions between metabolic, inflammatory, and immune pathways. Through its ability to regulate microglial activation, modulate cytokine production, influence astrocyte function, reduce oxidative stress, and reshape gut microbiota composition, the KD presents a promising therapeutic strategy for neuroinflammatory and neurodegenerative conditions. However, further research is essential to comprehensively elucidate the intricate relationship between the KD and neuroinflammation in the context of epileptogenesis. This understanding will be pivotal in the development of targeted and effective therapeutic strategies.

The limitations of this study include the inability to fully elucidate the specific role of NF‐κB signaling in mediating the therapeutic effects of the KD. Although our findings suggest that KD attenuates neuroinflammation via NF‐κB inhibition, the absence of targeted pathway manipulation (e.g., BAY 11‐7082 or NF‐κB overexpression models) limits causal claims. Future studies should integrate KD with targeted NF‐κB modulation, paralleling recent advancements in precision epilepsy therapeutics. A key focus of subsequent work will involve administering NF‐κB agonists (e.g., PMA) to KD‐treated animals to dissect whether pathway inhibition is necessary for the observed neuroprotection. Furthermore, this study primarily focuses on a single mechanism of action of the KD, without addressing other potential pathways that may also contribute to its effects on learning and memory. To address these limitations, we recommend conducting long‐term studies to evaluate the sustainability of the therapeutic effects and to establish the optimal duration of treatment. Future KD protocols should prioritize nutritional sufficiency unless explicitly targeting weight modulation, particularly in pediatric populations in where maintaining developmental trajectories is critical. Future research should aim to validate our findings in additional epilepsy models, including transgenic animals, to further substantiate the observed effects. Moreover, more studies are required to validate this hypothesis and offer solid experimental proof to aid in creating new therapeutic approaches targeting this pathway.

Our study has provided preliminary evidence that, following SE in rats, NF‐κB signaling is activated, resulting in the induction of neuroinflammation and structural damage in hippocampal neural circuits. KD intervention has been shown to inhibit NF‐κB signaling activation and neuroinflammation, whereas mitigating the structural damage in hippocampal circuits and cognitive deficits induced by SE. The anti‐inflammatory effects of KD in SE‐induced neural circuit damage and cognitive impairment suggest that KD may serve as a promising therapeutic strategy for SE and other neurological disorders with analogous pathologies.

## Author Contributions

**Wandi Wang:** writing – review and editing, writing – original draft, visualization, validation, software, methodology, investigation, data curation, conceptualization. **Lingman Wang:** writing – review and editing, writing – original draft, validation, methodology, investigation, data curation, conceptualization. **Chunxue Jiang:** writing – review and editing, validation, software, methodology, conceptualization. **Shengxuan Zhang:** software, methodology, visualization, investigation. **Chen Tan:** visualization, investigation. **Liqiong Peng:** writing – original draft, data curation. **Ran Ding:** visualization. **Bing Tian:** data curation. **Xiaojie Song:** writing – review editing, supervision, software, methodology, conceptualization. **Li Jiang:** writing – review and editing, supervision, software, methodology, conceptualization.

## Ethics Statement

The protocols were rigorously reviewed and approved by the Ethics Committee, Children's Hospital of Chongqing Medical University, under protocol CHCMU‐IACUC20240628009, thereby complying with both international and national regulations regarding animal experimentation.

## Consent

The authors have nothing to report.

## Conflicts of Interest

The authors declare no conflicts of interest.

## Data Availability

The data that support the findings of this study are available from the corresponding author upon reasonable request.
